# Paratyphoid a, Complicated with Purpura Haemorrhagica

**Published:** 1916-05

**Authors:** 


**Paratyphoid A, Complicated With Purpura Haemorrhagica.** E. Job, Soc. Med. des Hop., Nov. 12, 1915. Patient aged 12, diagnosis by pure culture of paratyphoid A, shortly after entrance to hospital Oct. 1. Moderate course till Oct. 21 when obstinate epistaxis began. Then purpuric spots, soon becoming general and followed by multiple haemorrhages. Death Oct. 27.

